# Design and application study of the key technical parameters of dense drilling for pressure relief in coal mine roofs: a case study

**DOI:** 10.1038/s41598-026-49581-1

**Published:** 2026-04-19

**Authors:** Junqiang Ma, Hongsheng Wang, Xuehua Li, Guowei Dong, Yuxin Yuan

**Affiliations:** 1 College of Energy and Mining Engineering, Xi’an University of Science and Technology, Xi’an, 710054 China; 2 School of Mines, China University of Mining and Technology, Xuzhou, 221116 Jiangsu China

**Keywords:** Dense drilling, Roof cutting, Pressure relief, Roadway stability, UDEC, Energy science and technology, Engineering, Solid Earth sciences

## Abstract

The lateral cantilever beam structure developed after hard roof workface mining can impose high stress on the adjacent roadway and coal pillar. As a result, the mining pressure of the adjacent roadway can drastically increase. In this study, the application of intensive drilling technology for roof weakening is examined in accordance with the principle of large and small structures of the surrounding rock. The mechanism of stress correspondence and the characteristics of the plastic area distribution around circular holes of different diameters are analyzed. A numerical model is developed using block discrete element calculation software (UDEC) considering the geological conditions of the 31,315 workface in the Chahasu Mine, and the effects of the height and angle of the roof cut on the collapse characteristics of the workface lateral cantilever structure and the mining pressure distribution in the roadway are investigated. As indicated by the study results, in a certain range, with increasing height and angle of the roof cut, the roof more significantly collapses and fills the goaf, contributing to roadway stability. As revealed by the field test results, dense drilling precracking can promote timely roof collapse, reducing the roadway stress level and ensuring roadway stability.

## Introduction

The roadway is the key gateway of the workface of underground coal mines. Roadway stability is closely correlated with the production safety and efficiency of the workface. The mode and strength of the surrounding rock support and the strength and stress of the surrounding rock are the major factors of roadway stability^1–3^. The effect of the surrounding rock stress on roadway stability encompasses the original stress and mining stress. The original stress imposes a major adverse effect on the excavation-stage roadway stability since the roadway layout has been optimized prior to excavation^4^. At this stage, the roadway surrounding rock is not significantly deformed, while favorable stability is attained. The mining stress exerts a major negative effect on the roadway stability in the workface at the mining stage, and the scale and distribution of the mining stress are correlated with the overburdened rock structure and the mining technology conditions of the workface^5–7^. In particular, the lateral cantilever beam structure formed by incomplete collapse of the immediate roof at the end of the workface and the dynamic adjustment in the surrounding rock stress caused by block rotary sinking after basic roof fracturing are important factors of roadway stability^8–10^. With the aim of ensuring the roadway stability during the course of production service, a variety of roadway stability control methods have been extensively developed, which mainly fall into four categories according to the following principles^11–15^: ① peripheral rock reinforcement; ② optimization of the roadway layout; ③ roof cutting to release the pressure; and ④ joint control. Over the past few years, as coal mining in China has increased in both intensity and depth, the number of roadways affected by intense mining has progressively increased, and such roadways exhibit high stability during the excavation period. Furthermore, the surrounding rock is drastically deformed after mining, some of which may be subjected to secondary repetitive mining or multiple repetitive mining activities during the production and service periods. The surrounding rock can hardly remain stable using surrounding rock reinforcement for this type of roadway only. In addition, the technical method of roof cutting should be employed to relieve the pressure and improve the roadway stress environment by adjusting the surrounding rock structure^1^. Specifically, part of the main roof rock stratum and the immediate roof rock stratum are precracked in advance through artificial intervention. Accordingly, these strata can fall in time to fill the goaf after workface mining, and a stable bearing structure can be formed as early as possible to optimize the peripheral rock stress of the roadway^16,17^.

Exploded polymerization directional blasting technology^18^ and directional hydraulic fracturing technology^19,20^ are the commonly used cutting roof relief technologies in Chinese coal mines. Exploded fusion directional blasting technology aims to alter the form of the explosive charge or the hole shape to control the detonation of explosives generated by the blast gas, thereby directing the stress wave action, as well as using the directional fusion effect to control the directional cracking of fractures; this technology represents an improvement in directional blasting technology^18^. This technology has been extensively applied in high-stress roadway surrounding rock stability control^16,21^, intense mining influence roadway surrounding rock control^22,23^ and roof cutting for gob-side entry^24–26^. However, the stress wave and vibration effects of blasting can induce coal-rock dynamic hazards^27^, which are potentially hazardous. Explosives in several mining areas in China have been more rigorously controlled, and the technical management and process of blasting and unloading are relatively complex. Hydraulic fracturing roof cutting technology involves injecting high-pressure fluid into the rock mass to generate stress that reaches the tensile strength of the rock, thereby creating fractures^28,29^, accompanied by water‒rock interactions^30,31^, thus destroying the structural integrity, reducing the rock mass strength, and facilitating the collapse of the roof formation in a timely manner. Over the past few years, Huang et al.^32–34^ developed theories and techniques related to hydraulic fracturing in coal rock masses and proposed hydraulic fracturing control theory and a complete set of technology systems for hard roofs. Kang et al.^35,36^ developed a system of directional drilling hydraulic fracturing technology for controlling the surrounding rock of coal mines, which has been popularized and applied in numerous mining areas. Yu et al.^37–39^ developed a hydraulic fracturing control technology system for the hard roofs of extrathick coal seams, which achieved satisfactory application results in addressing the problem of hard roof plate control in the Datong mining area. However, the hydraulic crack expansion direction depends on the maximum principal stress direction and is easily affected by the weak surfaces of rock layers and joint fissures. Thus, the initiation and expansion directions of hydraulic cracks are difficult to control^40–42^. In addition, the stress perturbation phenomenon at the crack tip during hydraulic fracture extension affects the characteristics of the stress distribution in the surrounding rock^43,44^. Furthermore, other technical methods can be used to control hard roof slabs in coal mines in China, including chain arm saw cutting roof relief technology^45–47^, composite blasting directional slit-making technology^48^ and instantaneous expansion cracking directional rock-breaking technology^49^. Furthermore, the instability of the roof induced by densely drilled holes is not only related to stress redistribution, but also closely associated with crack initiation and propagation around the boreholes, crack interaction, and the strain localization process^50,51^.

Compared to roof cutting and pressure relief technologies such as hydraulic fracturing and blasting, the dense borehole roof cutting and pressure relief technology offers advantages such as simpler processes, stronger adaptability, and better safety.

**Fig. 1:**
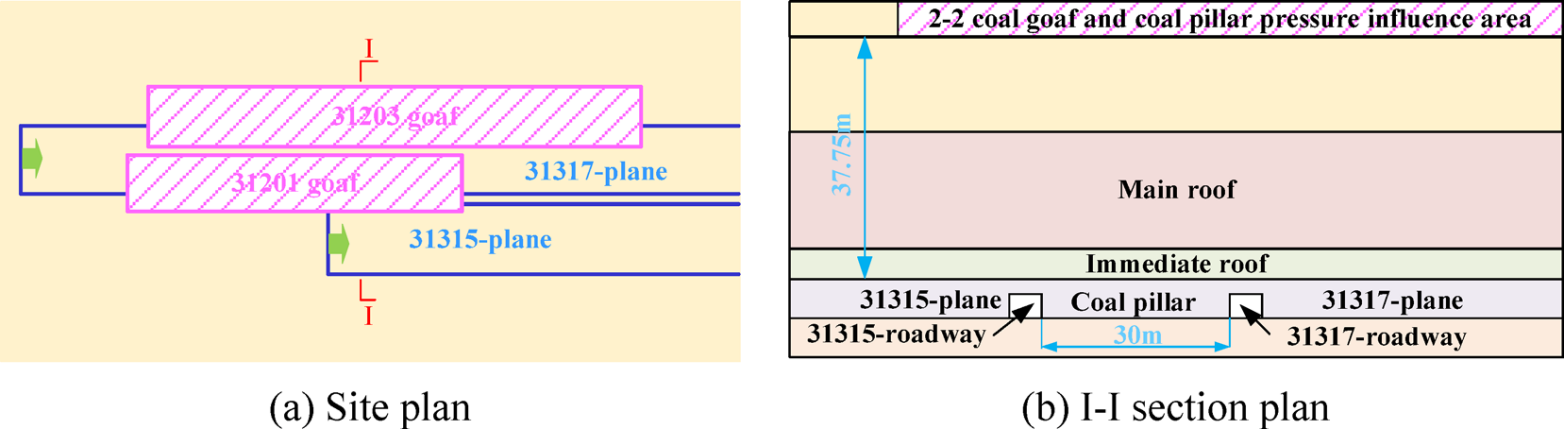
Mining engineering diagrams of the 31,315 workface.

The 31,315 workface on the east flank of the 3 − 1#coal seam and the subsequent workfaces are affected by the goaf and coal pillar pressures of the 2–2#coal seam, The 2–2#coal and 3 − 1#coal seams are the main coal seams of the Chahasu Coal Mine, and these coal seams exhibit a vertical spacing of 37.75 m. Figure 1 shows the 31,315 workface mining engineering plan and the sectional plan.

Moreover, the 31,315 workface and subsequent workfaces exhibit a roadway length over 5,000 m, inevitably passing through legacy coal pillars or the goaf of the upper coal seam. When the coal pillar of the 3 − 1 coal seam section is located in this area, the coal pillar is partially damaged, the roadway is seriously deformed, and frequent roof collapse accidents are triggered. Therefore, considering the abovementioned issues, following the principle of large and small structures of the surrounding rock, the idea of adopting large-diameter roof intensive drilling to unload the pressure for protecting the roadway is proposed in regard to stress adjustment of the surrounding rock, which is one of the control factors of the stability of the roadway surrounding rock, with the aim of satisfying the stability requirements of the adjacent roadways and ensuring safe production during the mining of the 31,315 workface and subsequent workfaces. Theoretical analysis is conducted to investigate the stress distribution characteristics and the plastic area range around circular drill holes. Moreover, numerical calculation is performed to analyze the breakage characteristics of the overburdened structure and the distribution of the mining pressure under different cutting heights and angles. Furthermore, an onsite industrial test is performed to investigate the effect of pressure relief resulting from intensive drilling to protect the roadway.

## Description of the geological conditions

The ground level of the 31,315 workface ranges from + 1280.8 to + 1364.3 m, and the floor level of the coal seam ranges from + 945.04 to + 960.00 m, with an average burial depth of 362 m. The 31,315 workface exhibits an advancing length of 4044.06 m, a width of 295.87 m, and an area of 1,196,500 m^2^. The 31,315 roadway is located in the 3 − 1 coal seam, occurring in the upper part of the Jurassic middle-lower Yan’an Formation. It pertains to the recoverable and stable coal seam within the entire area, serving as one of the main recoverable coal seams in the well field. The abovementioned seam is characterized by a relatively simple structure, with a thickness ranging from 5.65 ~ 6.60 m (an average thickness of 5.98 m) and a dip angle of 1°~3°.

**Fig. 2:**
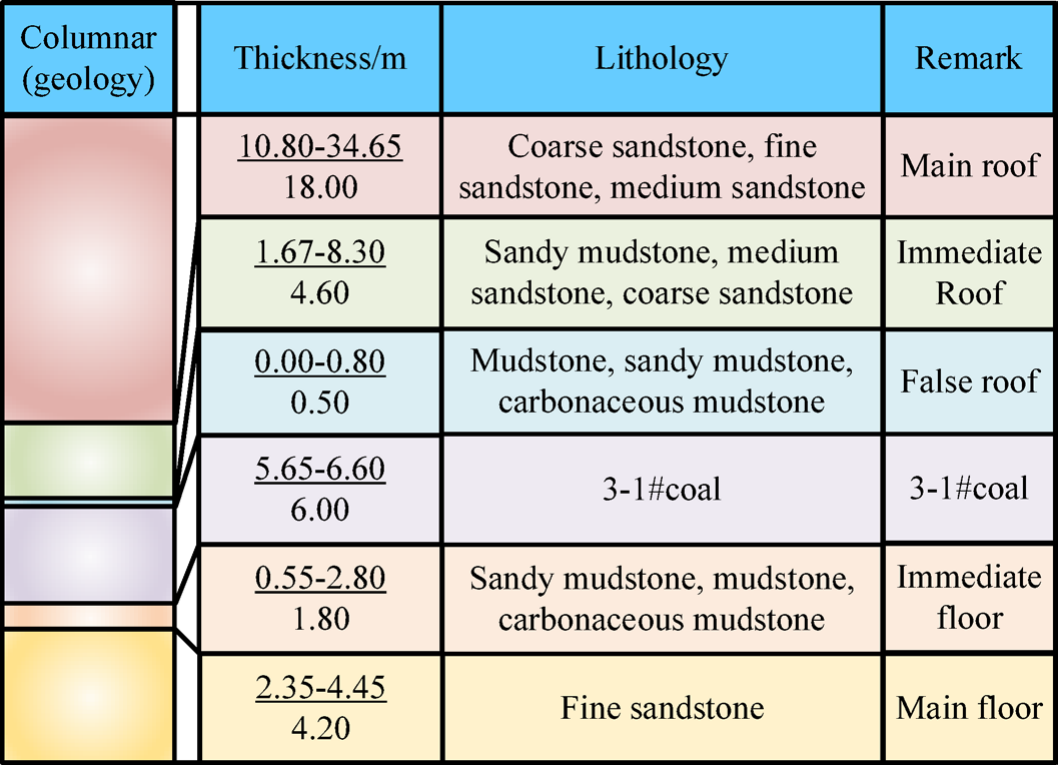
Composite column diagram of the rock strata in the 31,315 workface.

The 31,315 roadway of the Chahasu Coal Mine is excavated in line with the coal seam floor, leaving a top coal layer with a thickness of 1.5 m. As revealed by the columns of the drill holes close to the area of large deformation of the roadway perimeter rock, sandy mudstone, mudstone and carbonate mudstone, exhibiting an average thickness of 1.80 m, constitute the immediate floor rock strata in the area. In addition, fine sandstone comprises the main floor rock strata, with an average thickness of 4.20 m. Sandy mudstone constitutes the false roof, with an average thickness of 0.50 m. Furthermore, sandy mudstone, medium-grained sandstone and coarse-grained sandstone comprise the immediate roof rock strata, with an average thickness of 4.60 m. Medium-grained sandstone, fine sandstone and coarse sandstone constitute the main roof, with an average thickness of 18.0 m. Figure 2 shows the comprehensive stratigraphic column of the rock strata in the 31,315 workface.

**Fig. 3:**
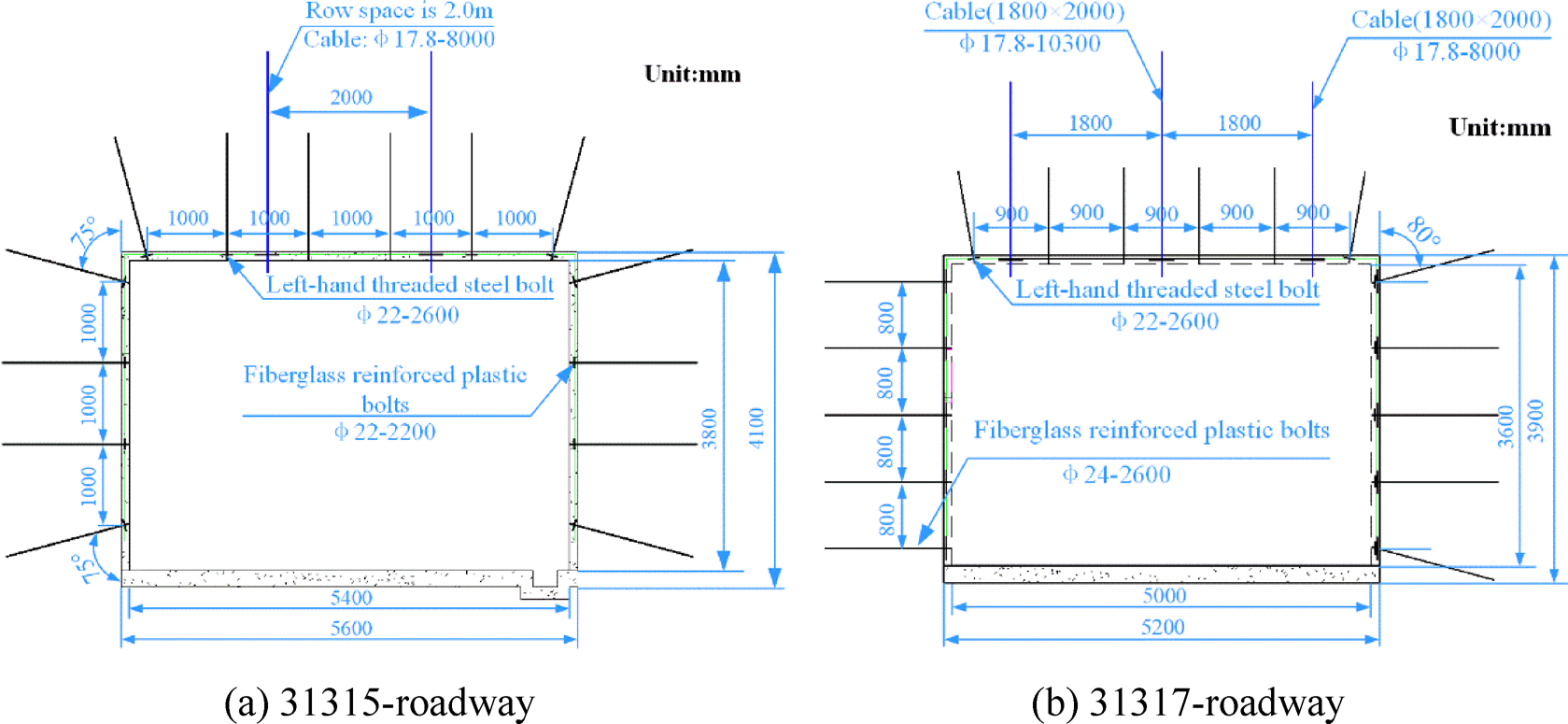
Support technical program of the 31,315 and 31,317 roadways.

The 31,315 roadway is supported with bolt mesh cables. Figure 3a shows the technical scheme of the support. The support parameters can be described as follows: ① the roof bolt adopts a φ22 × 2600 mm left-handed threaded steel bolt, exhibiting a spacing of 1000 mm×1000 mm. Moreover, φ22 × 2200 mm FRP anchors with a row spacing of 1000 mm×1000 mm constitute the mining side coal wall. The coal pillar side adopts φ22 × 2600 mm left-handed threaded steel anchors with a row spacing of 1000 mm×1000 mm. The corresponding bolt employs CK2360 resin anchoring agent, with an FRP anchoring force ≥ 78.4 kN and threaded steel anchoring force ≥ 100 kN. ② A φ17.8–8000 mm strand with a WD280-3.5 steel belt constitutes the anchor cable. The corresponding anchor cable employs 2 CK2360 resin anchors and 1 MSZ2360 resin anchor, with a preload force ≥ 180 kN. ③ The roof and coal pillar sides adopt φ6.5 mm welded reinforcement mesh with a grid size of 100 mm×100 mm. The mining side adopts plastic mesh, model HBPP30-30MS, with a grid of 40 mm×40 mm and a specification of 3,800 mm×10,000 mm.

The 31,317 roadway is supported with bolt mesh cables. Figure 3b shows the technical scheme of the support. The support parameters are as follows: ① the roof bolt adopts a φ22 × 2600 mm left-handed threaded steel bolt, with a spacing of 900 mm×1000 mm. The mining side coal wall contains φ24 × 2200 mm FRP anchors with a row spacing of 800 mm×1000 mm. The coal pillar side adopts φ22 × 2600 mm left-hand threaded steel anchors with a row spacing of 800 mm×1000 mm. The corresponding bolt employs CK2360 resin anchoring agent, with an FRP anchoring force ≥ 78.4 kN and a threaded steel anchoring force ≥ 100 kN. ② The anchor cable comprises φ17.8–10,000 mm and φ17.8–8000 mm strands with a WD280-3.5 steel belt. In the associated anchor cable, 2 CK2360 resin anchors and 1 MSZ2360 resin anchor are adopted, with a preload force ≥ 180 kN. ③ The roof and coal pillar sides adopt φ6.5 mm welded reinforcement mesh, with a grid size of 100 mm×100 mm. The mining side adopts plastic mesh, model HBPP30-30MS, exhibiting a grid of 40 mm×40 mm and a specification of 3800 mm×10,000 mm.

## Theoretical calculation of the dense drilling spacing

**Fig. 4:**
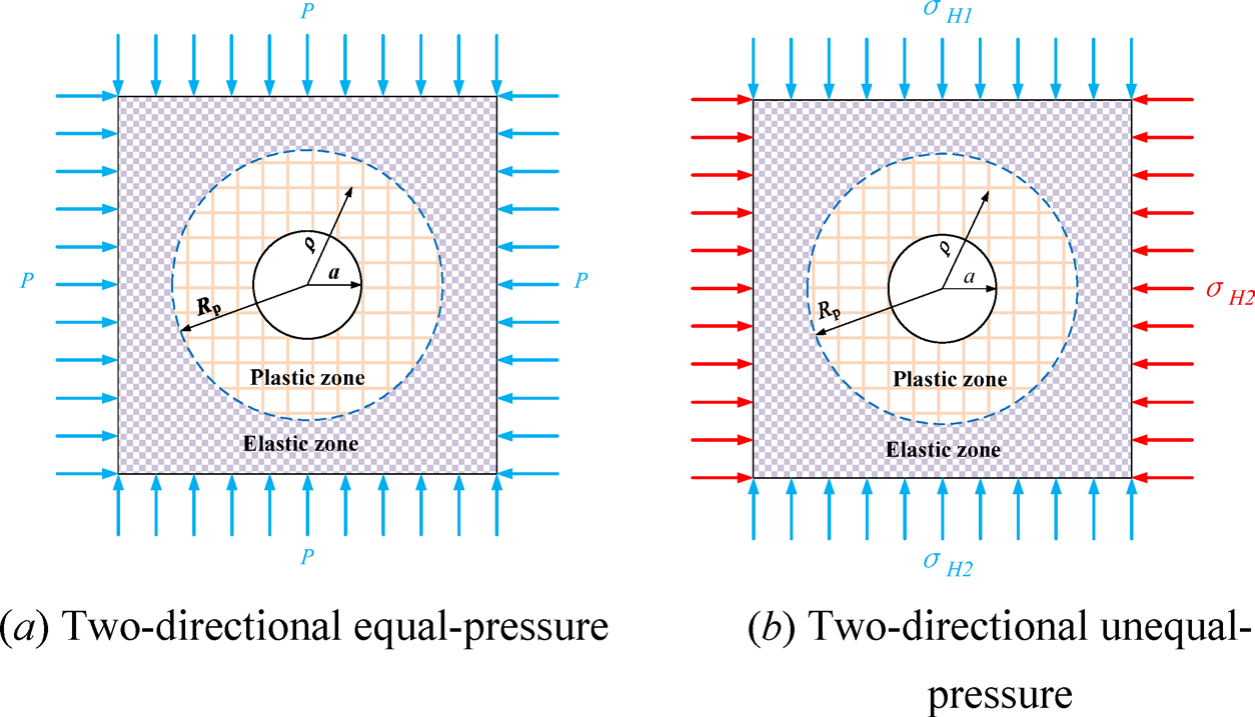
Schematic diagram of the forces around a circular borehole.

A circular borehole stress model is developed for investigating the mechanism of stress correspondence and the distribution range of the plastic area after borehole excavation. The rock mass where the drill holes are located is assumed to exhibit continuity, homogeneity, isotropy, and linear elasticity, and the surrounding rock exhibits an elastic state after excavation. Since the depth direction of the drill holes in this study is perpendicular to the roof plate, the horizontal stresses applied to the circular drill holes are simplified as the stress distribution around the drill holes after stress homogenization, as shown in Fig. 4^52,53^.

Since the depth of the roof borehole is notably larger than its cross-sectional size, the problem can be considered a plane strain issue. The stress component in the surrounding rock of a two-directional isobaric circular borehole in polar coordinates can be expressed following the theory of elasticity as follows^52,53^:

1 $$\left\{ \begin{gathered} {\sigma _\rho }{\mathrm{=}} - P(1 - \frac{{{a^2}}}{{{r^2}}}) \hfill \\ {\sigma _\varphi }{\mathrm{=}} - P(1+\frac{{{a^2}}}{{{r^2}}}) \hfill \\ \end{gathered} \right.$$

where *σ*_*ρ*_ is the radial stress at any point around the borehole, MPa; *σ*_*θ*_ denotes the tangential stress at any point around the borehole, MPa; *a* denotes the radius of the borehole, m; and *P* is the horizontal stress around the borehole, MPa.

Similarly, following the theory of elastic mechanics, the stress component in the rock surrounding a two-directional unequal-pressure circular borehole in polar coordinates satisfies the following^52,53^:

2 $$\left\{ \begin{gathered} {\sigma _\rho }=\frac{{{\sigma _H}_{2}}}{2}(1+\lambda )(1 - \frac{{{a^2}}}{{{r^2}}}) - \frac{{{\sigma _H}_{2}}}{2}(1 - \lambda )(1 - 4\frac{{{a^2}}}{{{r^2}}}+3\frac{{{a^4}}}{{{r^4}}})\cos 2\theta \hfill \\ {\sigma _\theta }=\frac{{{\sigma _H}_{2}}}{2}(1+\lambda )(1+\frac{{{a^2}}}{{{r^2}}})+\frac{{{\sigma _H}_{2}}}{2}(1 - \lambda )(1+3\frac{{{a^4}}}{{{r^4}}})\cos 2\theta \hfill \\ \end{gathered} \right.$$

where *σ*_*H*1_ and *σ*_*H*2_ are the maximum horizontal principal stress and the minimum horizontal principal stress around the borehole, respectively, MPa; and λ = *σ*_*H*1_/*σ*_*H*2_.

**Fig. 5:**
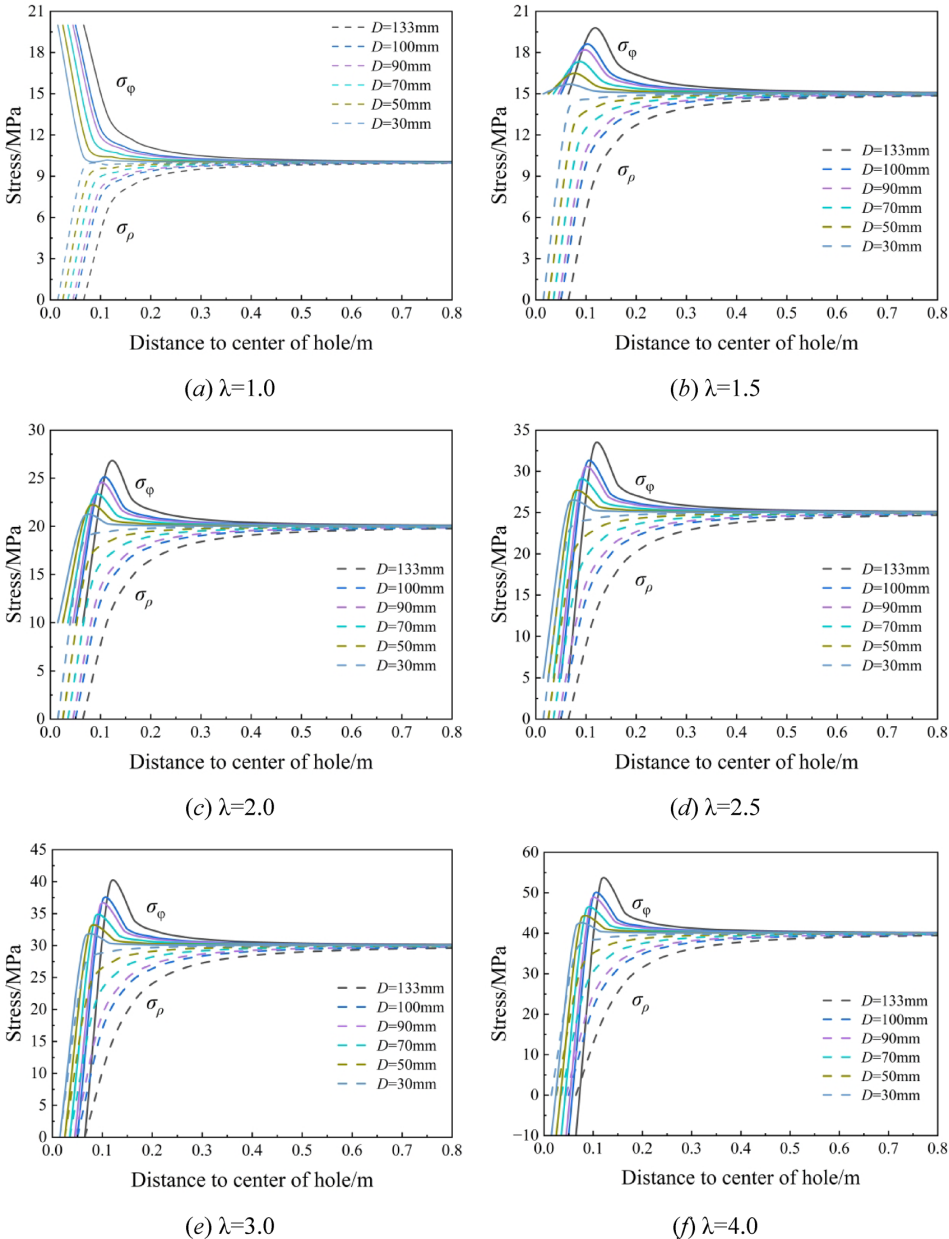
Stress distribution around circular holes of different diameters for various λ values.

From Eqs. (1) and (2), the distribution patterns of the radial stress *σ*_*ρ*_ and the tangential stress *σ*_*θ*_ around circular holes of different diameters can be obtained under varying horizontal stress ratios λ (Fig. 5). As shown in Fig. 5, the stress magnitude around the circular hole does not depend on the elastic constants *E* and *µ*, and the tangential stress σ_θ_ is the maximum stress. For *λ* = 1 (two-directional equal pressure), the maximum stress concentration factor *K* = 2.0 for the tangential stress *σ*_*θ*_, which does not depend on the circular hole diameter. Nevertheless, the effect of the elevated tangential stress area increases with increasing drill hole diameter. For *λ* > 1 (unequal pressure along two directions), the maximum stress concentration factor *K* of the tangential stress *σ*_*θ*_ increases with *λ* and *D*. When the diameter of the circular hole is D = 133 mm, *K* = 1.75 for *λ* = 1.5; *K* = 2.63 for *λ* = 2.0; *K* = 3.28 for *λ* = 2.5; *K* = 3. 94 for *λ* = 3.0; and *K* = 5.2 for *λ* = 4.0.

At the mining engineering site, the roadway surrounding rock is constantly subject to a complex stress field, which results from the effects of the original rock stress and mining stress. Guo et al.^52^ derived a boundary line equation of the plastic area of the surrounding rock in the nonuniform stress field of rock materials to determine the distribution range of the plastic area in the return mining roadway surrounding rock, which can be described as follows:

3 $$\begin{gathered} 9(1 - \lambda )^{2} \left( {\frac{a}{r}} \right)^{8} \left[ { - 12(1 - \lambda )^{2} + 6(1 - \lambda ^{2} )\cos 2\theta } \right]\left( {\frac{a}{r}} \right)^{6} + \left[ {10(1 - \lambda )^{2} \cos ^{2} 2\theta - 4(1 - \lambda )^{2} \sin ^{2} \varphi \cos ^{2} 2\theta } \right. \hfill \\ \left. { - 2(1 - \lambda )^{2} \sin ^{2} 2\theta - 4(1 - \lambda ^{2} )\cos 2\theta + (1 + \lambda )^{2} } \right]\left( {\frac{a}{r}} \right)^{4} + \left[ { - 4(1 - \pi )^{2} \cos 4\theta + 2(1 - \lambda ^{2} )\cos 2\theta } \right. \hfill \\ \left. - 4(1 - \lambda ^{2} )\sin ^{2} \varphi \cos 2\theta - \frac{{4C(\sigma _{{H1}} - \sigma _{{H2}} )\sin 2\varphi \cos 2\theta )}}{{\sigma _{{H2}}^{2} }} \right] \left( {\frac{a}{r}} \right)^{2} + \left[ {(1 - \lambda )^{2} - \frac{{\sin ^{2} \varphi (1 + \lambda + (2C\cos \varphi )}}{{(\sigma _{{H2}} \sin \varphi )^{2} }}} \right] = 0 \hfill \\ \end{gathered}$$

where *σ*_*H*1_ and *σ*_*H*2_ are the maximum horizontal principal stress and the minimum horizontal principal stress around the borehole, respectively, MPa; *a* denotes the radius of the borehole, mm; *r* and *θ* denote polar coordinates of any point on the plastic area boundary; *C* is the rock mass cohesive force, MPa; and *φ* is the internal friction angle, °.

**Fig. 6:**
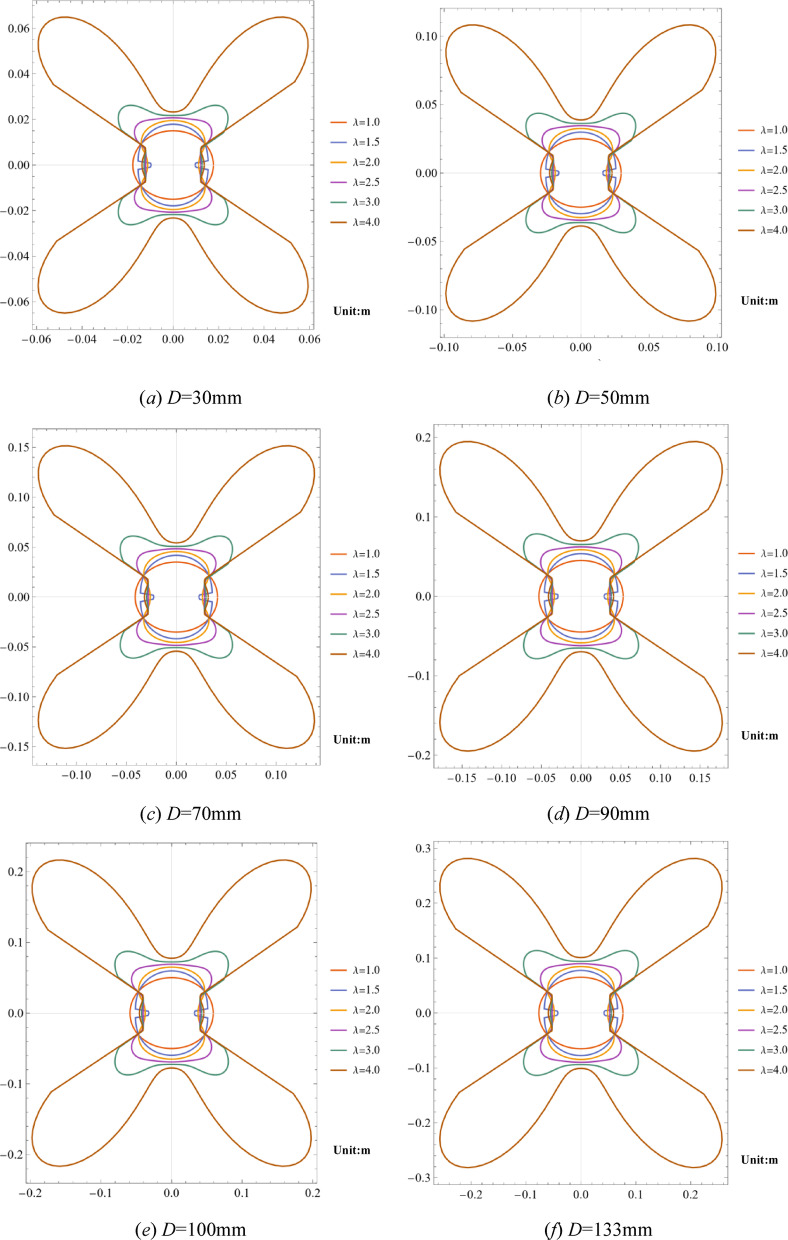
Distribution pattern of the plastic area around circular holes of different diameters under λ variation.

Equation (3) suggests that for rocky materials, *σ*_*H*1_ and *σ*_*H*2_ affect the size of the plastic area, whereas the shape of the plastic area is correlated with the ratio λ of *σ*_*H*1_/*σ*_*H*2_. The distribution of the plastic area around a circular hole within a range of diameter conditions when *C*, *φ* and *σ*_*H*2_ are set to 4.0 MPa, 30° and 12.0 MPa, respectively, is shown in Fig. 6. As depicted in the figure, the shape of the plastic area is circular for *λ* = 1. When *λ* increases, the plastic area is an ellipse with the lowest horizontal principal stress direction and the highest horizontal principal stress direction as the short and long axes, respectively. The plastic area becomes shaped as an X after *λ* increases to a certain value. When *λ* exceeds 3.0, the plastic area surrounding the circular hole expands rapidly and instantaneously. Moreover, as shown in Fig. 6a–f, the distribution range of the plastic area around the circular hole increases with increasing drill hole diameter *D* and *λ*. In the dense drilling pressure relief roadway technology process studied in this study, the construction of dense drilling holes is accomplished outside the effect range of the over support pressure of the working face, and this area is usually minimized under the effect of the mining stress in the working face, and *λ* is usually less than 2.0, resulting in the plastic area exhibiting a round or elliptical shape. The radius of the plastic area around the circular hole is *R*_p_≈19 mm and 2*R*_p_≈1.26*D* when the diameter of the circular hole is *D* = 30 mm under the condition of *λ* = 2.0; for *D* = 50 mm, *R*_p_≈41 mm and 2*R*_p_≈1.64*D*; for *D* = 70 mm, *R*_p_≈48 mm and 2*R*_p_≈1.37*D*; for *D* = 90 mm and 100 mm, *R*_p_≈60 mm and 2*R*_p_≈1.33*D*; and for *D* = 133 mm, *R*_p_≈81 mm and 2*R*_p_≈1.22*D*.

The results suggest that before the effect range of the workface over support pressure enters the intensive drilling construction area, the drill holes are only affected by the static pressure, and at this time, the distribution range of the plastic area around the hole (2*R*_p_) is approximately 1.22–1.64*D*. After the effect range of the workface over support pressure enters the intensive drilling construction area, the horizontal stress around the holes is redistributed under the effect of the mining stress, which leads to further expansion of the distribution range of the plastic area. The distribution range further increases. Wu et al.^54^ suggested that with the advancement of the workface to the stress monitoring position, the horizontal stress along the advancement direction of the workface rapidly increased to 1.5-3 times the initial stress. In addition, the variation in the horizontal stress perpendicular to the advancement direction of the workface is not significant. Thus, when the advancing direction of the working face coincides with the direction of the maximum horizontal principal stress, λ increases under the effect of mining. On this basis, the plastic area further expands around the round hole, and the shape of the plastic area ranges from a circle or an ellipse to an X shape.

In the above-described process, the rock mass around the borehole is gradually damaged along the direction of departure from the center of the circular hole, which gradually causes a decrease in the distance between the boundaries of the plastic areas around the adjacent boreholes and thus reduces the strength of the rock layer in the top plate. Under the condition of *λ* = 4.0, when the diameter of the circular hole is *D* = 30 mm, the radius of the plastic area around the circular hole is *R*_p_≈80 mm and 2*R*_p_≈5.33*D*; for *D* = 50 mm, *R*_p_≈120 mm and 2*R*_p_≈4.8*D*; for *D* = 70 mm, *R*_p_≈150 mm and 2*Rp* ≈ 4.28*D*; for *D* = 90 mm, *R*_p_≈200 mm and 2*R*_p_≈4.44*D*; for *D* = 70 mm, *R*_p_≈200 mm and 2*R*_p_≈4.44*D*; and for *D* = 90 mm, *R*_p_≈200 mm and 2*R*_p_≈4.44*D*. For D = 100 mm, *R*_p_≈220 mm and 2*R*_p_≈4.4*D*; and for D = 133 mm, *R*_p_≈280 mm and 2*R*_p_≈4.21*D*. This indicates that after the effect range of the over support pressure of the working face enters the construction area of the intensive drilling holes, the holes are affected by the mining stress, and the distribution range of the plastic area around the circular holes at this time (2*R*_p_) is approximately 4.21–5.33*D*. Accordingly, the distribution range of the intensive drilling holes is approximately 4.21–5.33*D*. Thus, the spacing *L* of the intensive drilling holes can be set according to *L* = 8 ~ 10*D*.

## Design of the roof-cut height and angle according to discrete element numerical simulation of blocks

### Numerical computational modeling

**Fig. 7:**
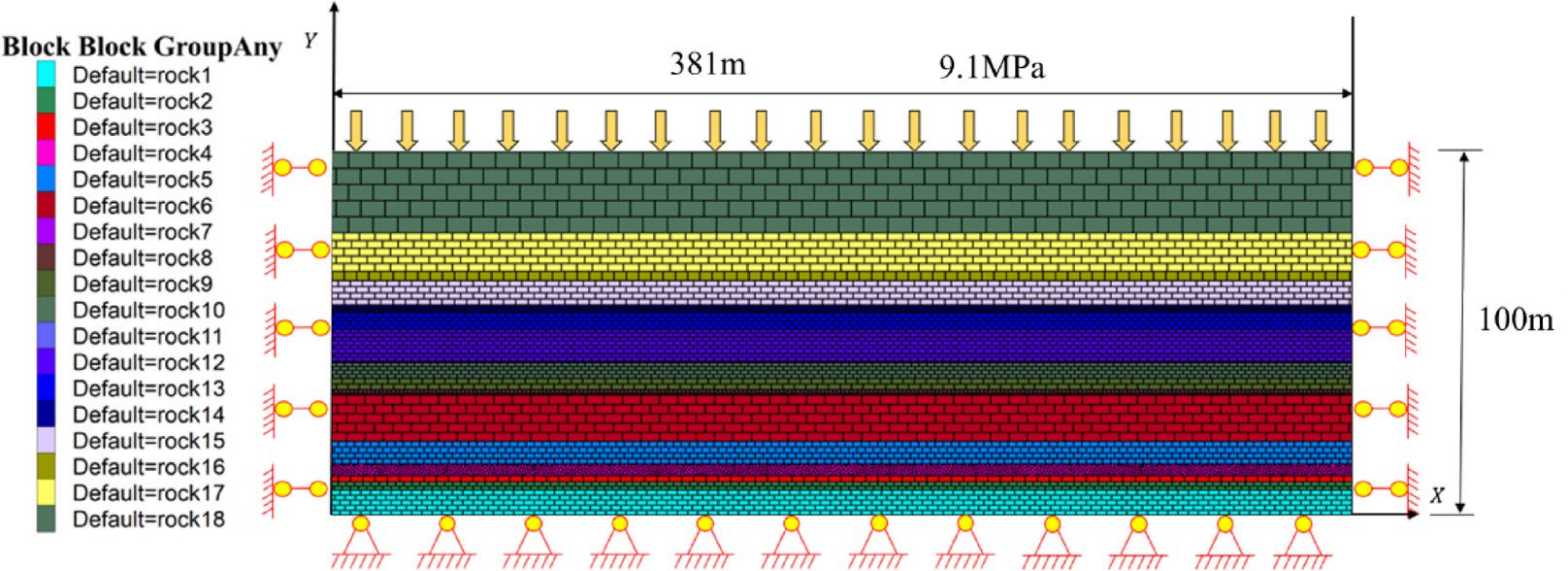
Schematic diagram of the numerical calculation model.

To explore the influence mechanism of roof-cut height and angle on the decompression effect of roof collapse features, combined with the field conditions, a numerical calculation model of roof-cutting decompression is built in this study considering the engineering geological conditions of the 31,315 workface in the Chahasu Mine using block discrete element numerical computation software (UDEC), with a size of 381 m×100 m. Moreover, the Mohr‒Coulomb model is adopted for the rock stratum principal structure, and the bottom and left and right boundaries of the model are constrained by limited displacement constraints. In addition, a vertical stress of 9.1 MPa is applied to the upper boundary of the model to simulate the gravity stress of the overlying rock strata, schematic diagram of the numerical calculation model as shown in Fig. 7. Rock samples collected from the site were processed into standard test blocks, and their compressive strength, elastic modulus, and tensile strength parameters were obtained through laboratory testing. Engineering-scale rock mass strength parameters were derived using the rock mass strength reduction method. Subsequently, meter-scale uniaxial compression and Brazilian splitting models were established in UDEC, and the input parameters of the numerical model were calibrated through a trial-and-error method. Table 1 lists the mechanical parameters of the various rock strata, and Table 2 lists the block and contact mechanical properties of the UDEC model.

**Table 1:** Coal rock mass mechanical and physical parameters.

Lithology	Uniaxial compressive strength/MPa	Elastic modulus/GPa	Poisson ratio	Density kg/m^3^	Internal friction angle /°	Cohesion /MPa	Tensile strength /MPa
Siltstone	47.6	17.8	0.24	2680	31	2.3	4.76
Sandy mudstone	27.8	14.1	0.26	2450	27	1.8	2.78
2-2#coal	12	1.5	0.32	1600	20	0.7	1.2
Sandy mudstone	27.8	14.1	0.26	2450	27	1.8	2.78
Mudstone	22.1	10	0.29	1900	25	1.4	2.21
Siltstone	47.6	17.8	0.24	2680	31	2.3	4.76
Sandy mudstone	27.8	14.1	0.26	2450	27	1.8	2.78
3-1#coal	12	1.5	0.32	1600	20	0.7	1.2
Sandy mudstone	27.8	14.1	0.26	2450	27	1.8	2.78
Fine sandstone	68.8	22.5	0.22	2800	29	2.6	6.88

In the simulation process, model excavation is completed in line with the following steps after the model is first balanced. Step 1: The 31,201 workface is excavated to form an overburdened coal seam working face hollow area and provide the fixed support stress in the hollow area. Step 2: The 31,315 roadway is excavated after overburdened rock movement, and stress adjustment tends to stabilize after the excavation of the 31,201 workface. Step 3: Drill holes are excavated after the disturbances generated during roadway excavation are balanced. Step 4: The drill holes are fully excavated, the 31,315 workface is excavated, and the transport and collapse patterns of the overburdened rock are observed.

**Table 2:** UDEC model block and contact mechanical property parameters.

Lithology	Elastic modulus /GPa	Density kg/m^3^	Internal friction angle/°	Cohesion/MPa	Tensile strength/MPa	k_*n*_ GPa/m	k_s_ GPa/m
Siltstone	17.8	2680	33	2.3	4.76	215.9	84.2
Sandy mudstone	14.1	2450	30	1.8	2.78	187.8	75.1
2-2#coal	1.5	1600	28	0.7	1.2	86.2	34.5
Sandy mudstone	14.1	2450	30	1.8	2.78	187.8	75.1
Mudstone	10	1900	28	1.4	2.21	112.4	45
Siltstone	17.8	2680	33	2.3	4.76	215.9	84.2
Sandy mudstone	14.1	2450	30	1.8	2.78	187.8	75.1
3-1#coal	1.5	1600	28	0.7	1.2	86.2	34.5
Sandy mudstone	14.1	2450	30	1.8	2.78	187.8	75.1
Fine sandstone	22.5	2800	31	2.6	6.88	286.2	114.5

### Effect of the roof-cut height on the roadway stability

**Fig. 8:**
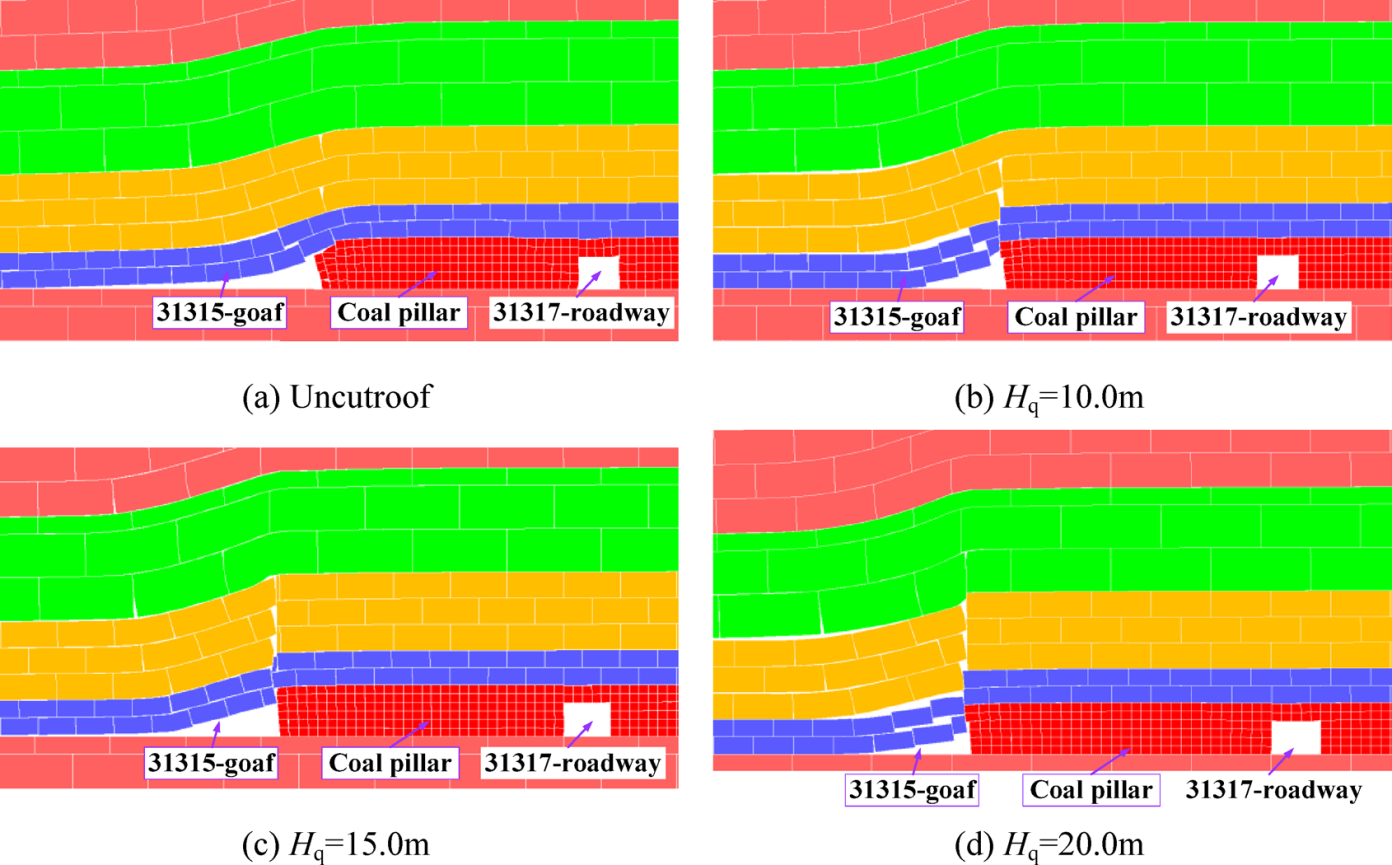
Fracture morphology of the lateral roof plate of the workface with roof-cut height variation.

In this section, the collapse characteristics of the lateral roof rock stratum and the stress distribution in the coal pillar of the section after mining are simulated under no roof-cutting and various roof-cut heights (including 10 m, 15 m, and 20 m), with the aim of determining the effect of the roof-cut height on the cantilever structure of the lateral roof rock stratum and the coal pillar stress distribution. Figure 8 shows the fracture form and collapse characteristics of the lateral roof rock strata in the 31,315 workface roadway after mining under a range of roof-cut height values. As depicted in the figure, under roof-cut heights of 10 m, 15 m, and 20 m, the lateral roof rock strata of the 31,315 workface after mining collapse follow the direction of the roof-cutting line accordingly and subsequently fill the goaf space. In addition, the fractured rock mass resulting from the collapse of the roof rock strata forms an effective support structure in the goaf space, suppressing the rotational sinking of the incompletely fractured rock mass of the roof at a high level, and a stable load-bearing structure is established in a timely manner. Moreover, as shown in Fig. 8, when the roof-cut height increases, the direct roof rock strata collapse more sufficiently to fill the goaf space. As a result, a stable bearing structure can be more easily formed, increasing the stability of the peripheral rock of the roadway. Since the implementation of artificial roof-cutting measures leads to a decrease in the strength of the roof rock strata at different levels, the roof rock strata collapse in a timely manner, and the goaf space becomes filled after workface mining. In addition, a stable bearing structure is generated in time to inhibit the rotary subsidence of the roof rock strata, improving the stress environment of the roadway and the coal pillar. Under the different roof-cutting heights, the collapse pattern of the roof rock strata and the filling level of the goaf vary, such that the optimization level of the roadway and coal pillar stress environment by the artificial roof-cutting measures differs.

**Fig. 9:**
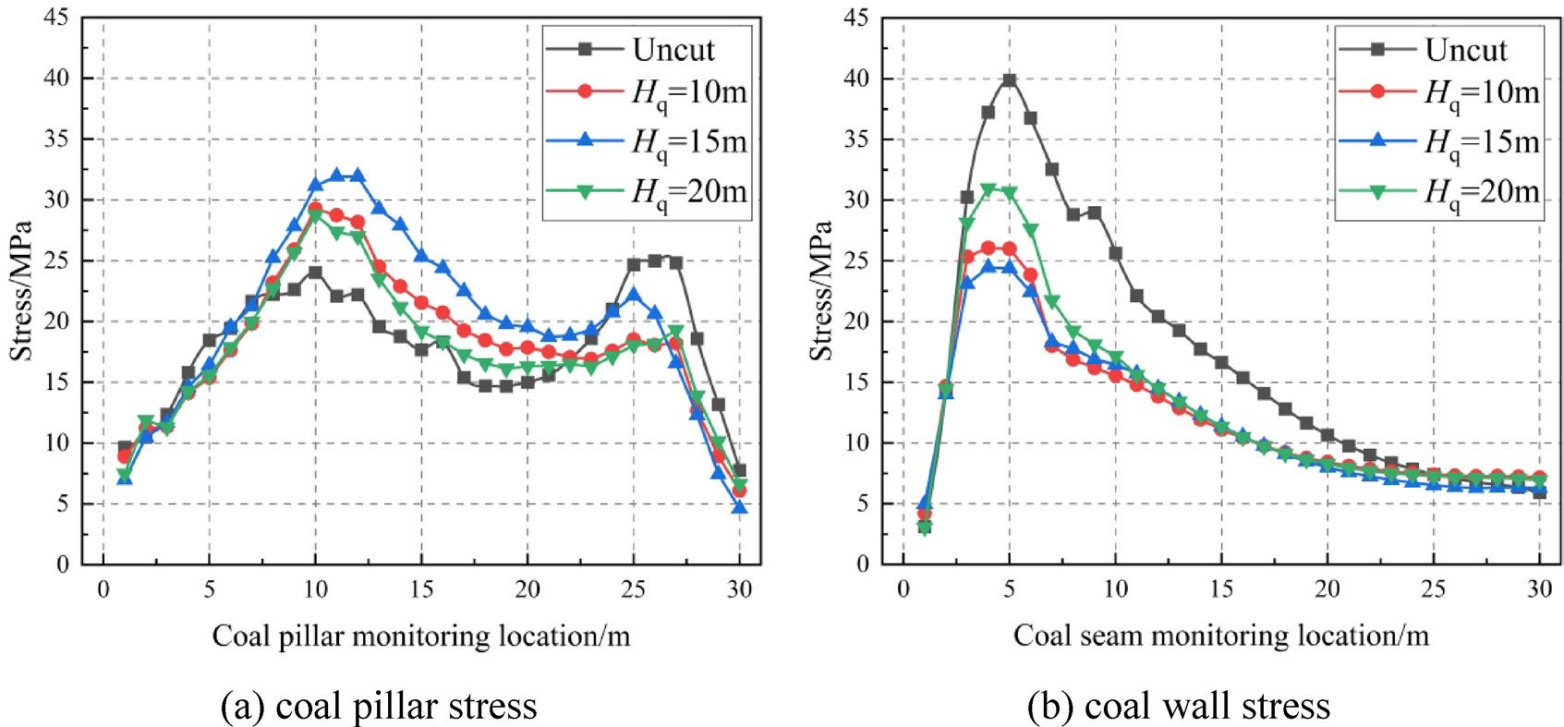
Vertical stress variation trend of the coal pillar and solid coal under roof-cut height variation.

Accordingly, to reveal the effect of the roof-cut height on the surrounding rock stress of the roadway, the change trend of the vertical stress on the two sides of the roadway when the roof-cut height is varied is investigated in this section, as shown in Fig. 9. The peak vertical stresses on the coal pillar side and solid coal side of the 31,317 roadway after 31,315 workface mining are 25.00 and 39.82 MPa, respectively, without roof cutting. Under roof-cut heights of 10 m, 15 m, and 25 m, the peak vertical stresses on the coal pillar side of the 31,317 roadway reach 18.53 MPa, 22.14 MPa and 19.31 MPa, respectively, after 31,315 workface mining, which are 6.47 MPa, 2.86 MPa and 5.69 MPa lower, respectively, than those when the roof is not cut. The peak vertical stresses on the solid coal side of the 31,317 roadway are 26.07 MPa, 24.45 MPa, and 30.97 MPa, respectively, which are 13.75 MPa, 15.37 MPa, and 8.85 MPa lower, respectively, than those without roof cutting. The above results indicate that under a roof-cut height less than 15 m, the workface precracking roof can effectively alleviate the stress load carried by the neighboring roadway and the coal pillar, such that a satisfactory roof-cutting effect can be achieved, and the pressure can be released to protect the roadway.

**Fig. 10:**
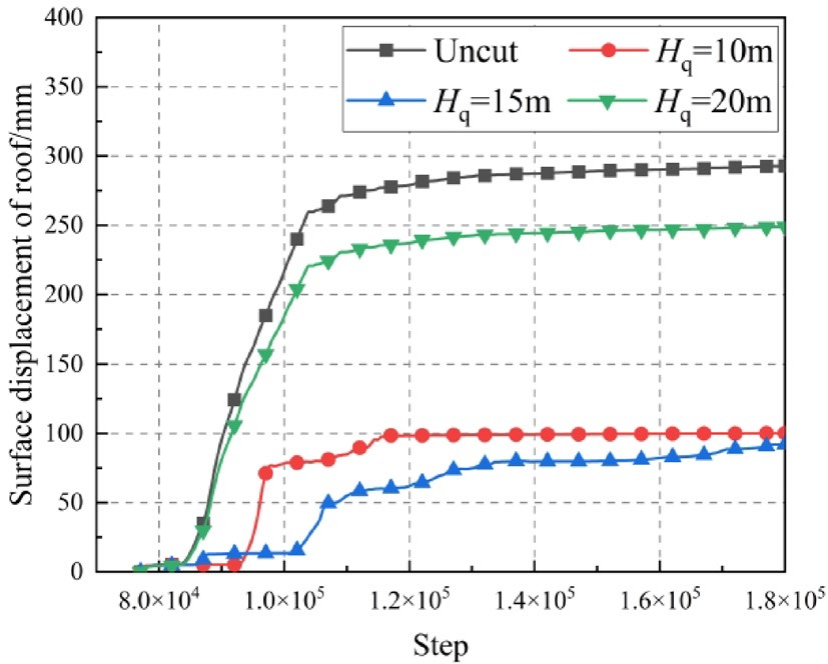
Variation rule of roof displacement of the 31,317 roadway with variation in the roof-cut height.

Figure 10 shows the variation rule of roof displacement of the 31,317 roadway in the mining process of the 31,315 workface under the different cutting height conditions. As shown in Fig. 9, under roof-cut heights of 10 m, 15 m, and 25 m, after 31,315 workface mining, the subsidence of the roof plate of the 31,317 lane is 92.4 mm, 100.3 mm, and 249.3 mm, respectively, which is 68.5%, 65.8%, and 15% less, respectively, than that when the roof is not cut, which is 293.3 mm. Notably, significant differences exist in the variation rule of roadway roof displacement under the different roof-cut heights. As the height increases, the roof deformation amount and the deformation speed increase, and the different roof-cut heights are correlated with the lateral roof breakage characteristics and the rock mass collapsing process, determining the characteristics of the stress distribution and the roadway roof decompression effect, as well as generating different evolution trends of roof deformation.

### Effect of the roof-cut angle on the roadway stability

In this section, the collapse characteristics of the lateral roof rock strata and the stress distribution in the coal pillar after workface mining are simulated under no roof-cutting and various roof-cut angles of 0°, 15°, 15° and 25° to reveal the effect of the roof-cut angle on the cantilever structure of the lateral roof rock strata and the stress distribution in the coal pillar of the workface.

**Fig. 11:**
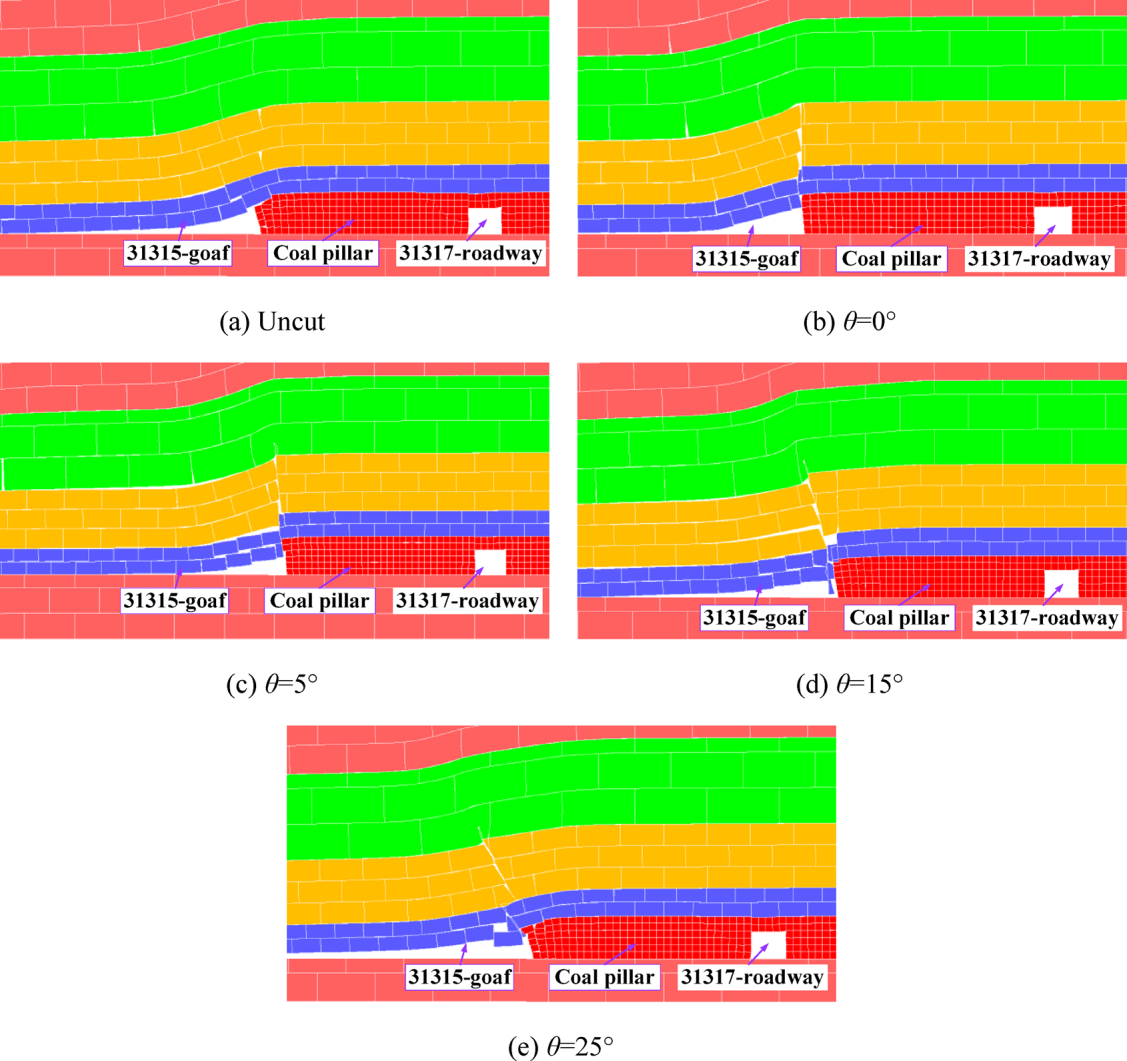
Fracture morphology of the lateral roof plate of the workface with roof-cutting angle variation.

Figure 11 shows the fracture morphology and collapse characteristics of the lateral roof rock strata of the roadway under a range of roof-cut angle conditions after 31,315 workface mining. At roof-cut angles of 0° and 15°, the length of the lateral cantilever structure in the goaf after 31,315 mining notably decreases, obviously facilitating control of the stability of the surrounding rock of the roadway. At a roof-cut angle of 25°, the lateral cantilever length of the goaf after workface mining increases, impeding control of the stability of the surrounding rock in the roadway. As revealed by these results, when the roof-cut angle is increased, the length of the lateral cantilever structure of the goaf after 31,315 workface mining decreases, and better filling of the goaf space and a timely support role are obtained. However, from the perspectives of the coal pillar stress distribution and coal pillar bearing analysis, the increase in the roof-cut angle does not facilitate control of the stability of the roadway surrounding rock. Specifically, in a certain range, the higher the roof-cut angle is, the higher the stability of the perimeter rock of the roadway; when the roof-cut angle exceeds a certain critical value, the effect of roadway perimeter rock stress relief is difficult to achieve. The strength of the roof rock strata is reduced to different degrees after the implementation of artificial roof-cutting measures. Thus, the roof rock strata can collapse and fill the mining goaf in a timely manner after workface mining, and a stable bearing structure can be formed in a timely manner to inhibit the rotary subsidence of the roof rock strata, improving the stress environment of the coal pillars and roadway. However, at the different roof-cut angles, the collapse pattern of the roof rock strata and the filling degree of the goaf differ, such that the optimization degree of the stress environment of the roadway and the coal pillar achieved by the adoption of artificial roof-cutting measures also varies.

**Fig. 12:**
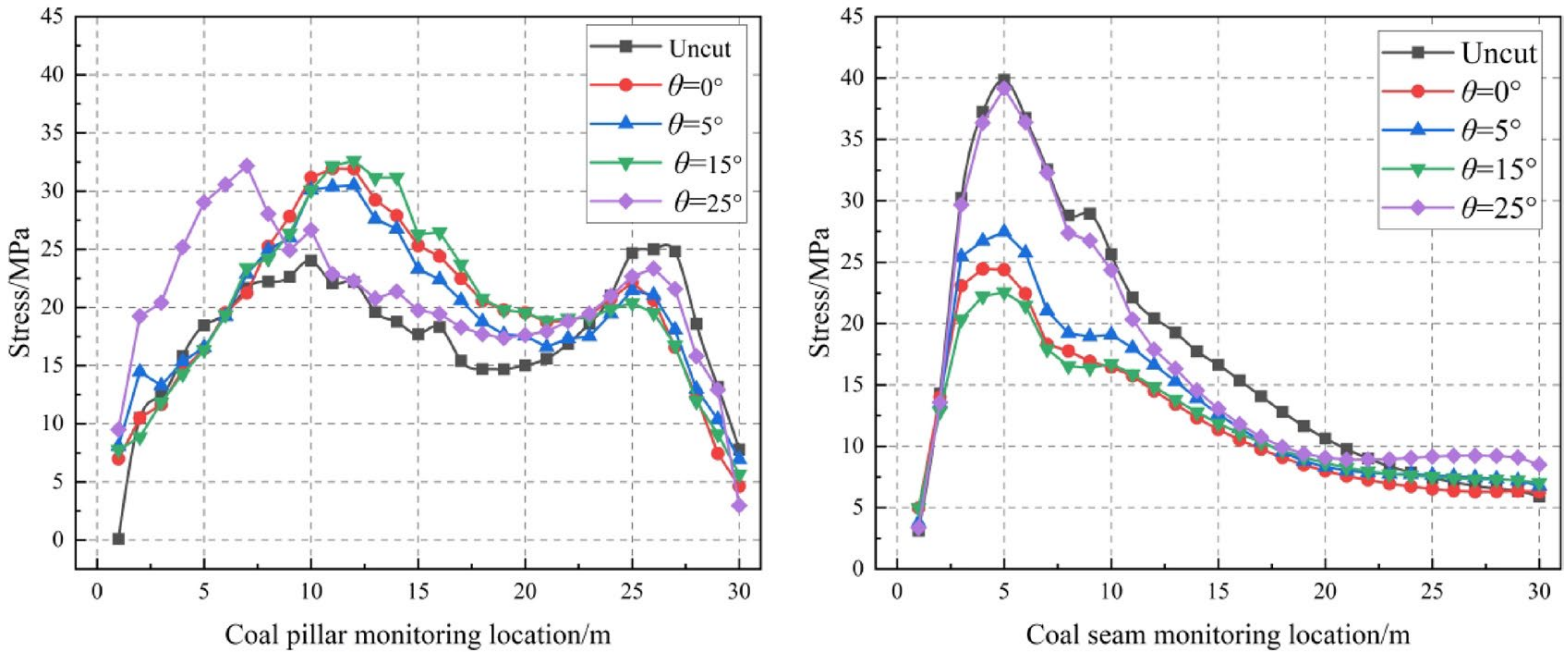
Vertical stress variation rule of the coal pillar and solid coal side under roof-cut angle variation.

Thus, the change rule of the vertical stress on the two sides of the roadway at the different roof-cut angles is investigated in this section, as shown in Fig. 12, to reveal the effect of the roof-cut angle on the surrounding rock stress of the roadway. When roof cutting is not implemented, the peak vertical stresses on the coal pillar side and solid coal side of the 31,317 roadway after 31,315 workface mining are 25.00 and 39.86 MPa, respectively. At roof-cut angles of 0°, 5°, 15° and 25°, the peak vertical stresses on the coal pillar side of the 31,317 lane after 31,315 workface mining are 22.14 MPa, 21.47 MPa, 20.38 MPa and 23.34 MPa, respectively. The peak vertical stresses at 30 m in the shallow area on the coal side of the 31,317 roadway are 22.14 MPa, 27.47 MPa, 22.55 MPa and 39.16 MPa, respectively. It is considered that when the roof-cut angle is less than 15°, the working face can effectively alleviate the stress load borne by the neighboring roadway and coal pillar through the workface above the pre-cracked roof plate, such that a favorable roof-cutting effect can be achieved, and the pressure can be unloaded to protect the roadway.

**Fig. 13:**
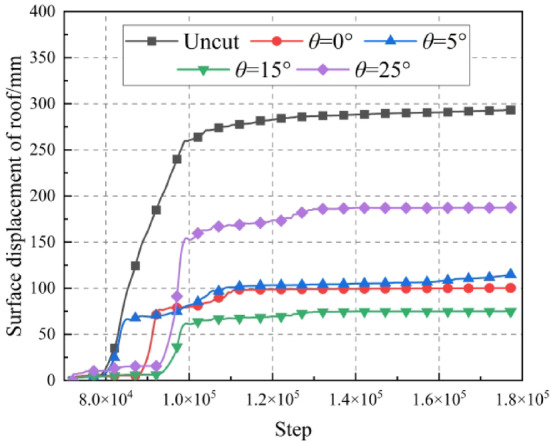
Displacement variation rule of the roof of the 31,317 roadway under roof-cut angle variation.

Figure 13 shows the variation rule of the 31,317 roadway roof displacement during 31,315 workface mining at different roof-cut angles. As shown in Fig. 13, at roof-cut angles of 0°, 5°, 15°, and 25°, after 31,315 workface mining, the roof subsidence of the 31,317 roadway is 100.3 mm, 114.5 mm, 75.06 mm, and 187.65 mm, respectively, which is 65.8%, 60.9%, 74.4%, and 36% less, respectively, than that when the roof is not cut. The above results suggest that when the angle of the roof-cutting drill holes is below 15°, with increasing angle of the roof-cutting drill holes, the roof plate deformation amount and speed in the roadway test area rapidly decline. Moreover, when the angle of the roof-cutting drill holes is 15°, the roof plate sinks the least, and roof-cutting decompression optimally protects the neighboring roadway. Furthermore, when the roof-cutting drill holes exhibit an angle over 15°, the deformation amount and the deformation speed of the roof plate continue to increase, hindering control of the roadway stability.

## Analysis of the engineering application effect

### Technical program for pressure relief with dense drilling

Following the calculation method of the intensive drill hole spacing and the numerical simulation results of the roof-cut height and angle, based on the engineering geology and production technology conditions of the 31,315 workface of the Chahasu Coal Mine, the results indicate that the intensive drill holes in the roof exhibit a diameter of 133 mm, the drill holes exhibit a depth of 15.0 m, and the angle of the holes is perpendicular to the roof plate.

**Fig. 14:**
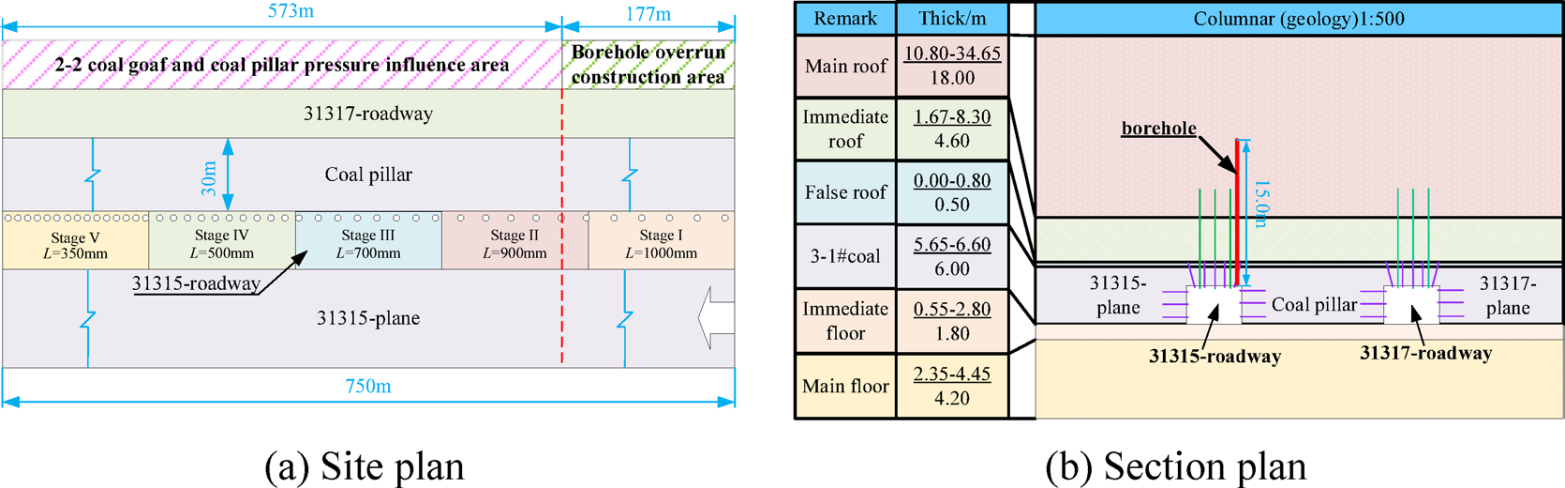
Schematic diagram of the large-diameter dense drilling arrangement.

The total length of the roadway in the drilling construction area is 750 m, of which 573 m occurs in the pressure-affected area of the 2–2 coal mining area. To ensure the decompression effect of the large-diameter drill holes, considering the amount of drilling construction work, theoretical calculations are conducted at five levels of the drilling spacing. Figure 14 shows the drilling arrangement technology program. The length of each stage of the drilling construction area roadway length reaches 150 m, and the spacing of the drilling holes reaches 1000 mm, 900 mm, 700 mm, 500 mm, and 350 mm.

### Application effect

**Fig. 15:**
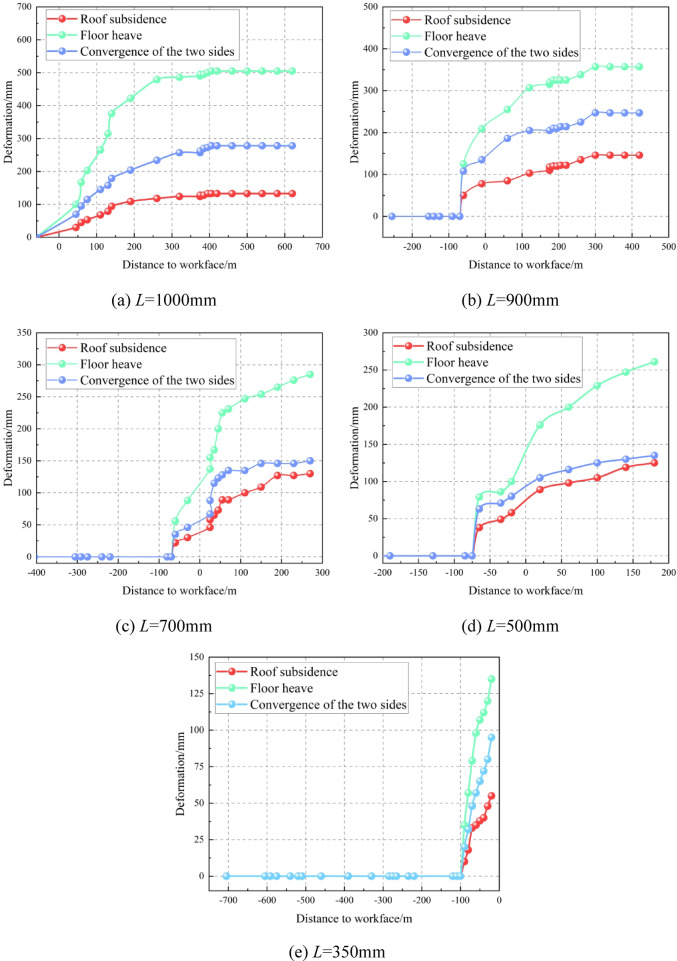
Surrounding rock deformation of the 31,317 roadway for the different dense drilling spacings.

In the mining process of the 31,315 workface, the surface deformation of the roadway peripheral rock of the 31,317 lane is monitored by a measuring station (Fig. 15). As depicted in the figure, in the process of advancing the 31,315 workface, the measuring station of the 31,317 roadway starts to record roadway deformation under the effect of the overpassing stress of the 31,315 workface when it passes the workface by 50–100 m, the roadway deformation basically remains stable when it lags behind the workface by 200 m, and the bottom heave is the greatest at the various stations, followed by the shifting amount of the two sides, with the minimum roof plate subsidence.

The floor subsidence amount at the location of the measuring station in the area where the spacing of the roof plate intensive drilling holes is 1000 mm, 900 mm, 700 mm, 500 mm and 350 mm is 505 mm, 357 mm, 285 mm, 261 mm and 135 mm, respectively, which shows that the larger the spacing of the intensive drilling holes in the area of the measuring station, the larger the amount of floor subsidence of the 31,317 roadway is. This occurs because the larger the dense drilling holes in the roof plate are, the lower the penetration degree of the artificial weakening area produced by the dense drilling holes and the lower the weakening effect of the dense drilling holes on the roof rock strata. Moreover, the cantilever structure formed on the side of the coal pillar after 31,315 face mining cannot collapse in time. Thus, the larger the spacing of the dense drilling holes is, the higher the stresses inside the coal pillar and at the location of the 31,317 roadway, resulting in more considerable bottom heave in the 31,317 roadway.

## Discussion

### The connotation of dense drilling roof cutting and pressure relief technology

When the coal seam is extracted from the workface, with the hydraulic support moving forward, the immediate roof rock strata collapse and fill the mining area to form a collapsed area. As the workface advances to the limit span of the collapse of the main roof rock strata, the main roof is fractured to form a fracture area, and under the extrusion of the horizontal stress, the rock blocks in the fracture area combine form a voussoir beam structure^55^.

**Fig. 16:**
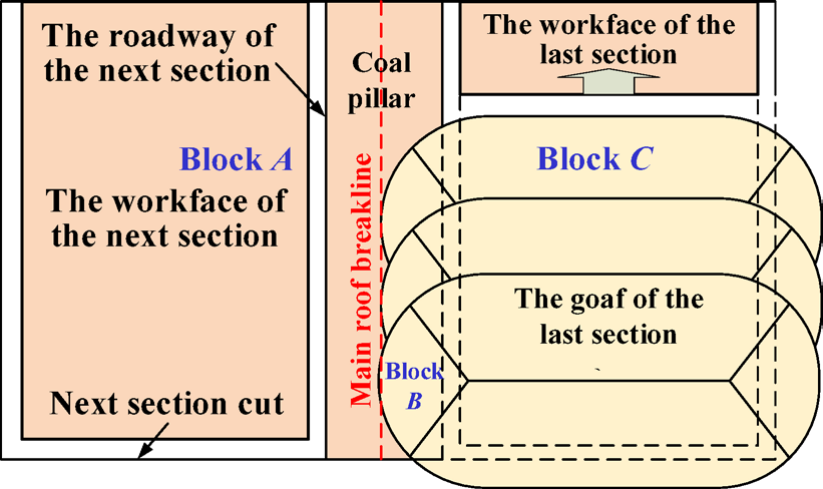
Roadway surrounding rock structure relationship plan.

The author’s research team proposed the roadway perimeter rock big-small structure principle^56^. The big structure refers to the larger range of the rock structure of the roadway peripheral rock, which comprises the top coal, immediate roof, main roof, and main load rock strata. The small structure refers to the shallow peripheral rock of the roadway and the anchoring structure of the combination of the formation of the anchored solid. As shown in Fig. 16, in the last workface mining process, with the advancement of the workface, arc-shaped triangular plate rock block *B* is formed in the coal pillar above the main roof rock strata fracture, one end of rock block *B* is subjected to rotary sinking in the goaf after touching the gangue, and the other end of the coal pillar or the lower section of the coal wall above is fractured. Over time, the rotary sinking process of rock block *B* stabilizes after rock block *A*, and rock blocks B and *C* exhibit mutual occlusion to form a stable articulated structure.

**Fig. 17:**
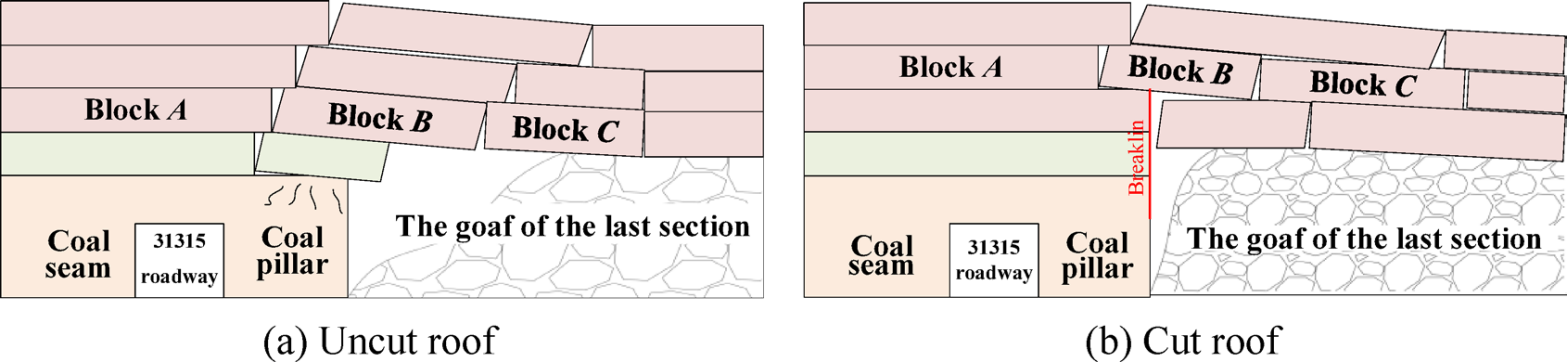
Section of the roadway surrounding rock structure with and without roof cutting.

As shown in Fig. 17a, under the effect of mining, the weight of the lateral cantilever beams formed by the incomplete collapse of the direct roof of the large structure and the overburden loads transmitted downward through key block *B* are borne by the small structure, while the rotational subsidence of key block *B* of the basic roof of the large structure also leads to an increase in the load of the small structure. However, the magnitude of the loads on the small structure of the surrounding rock is correlated with the length and thickness of the direct roof cantilever beams, the location of the fracture of the basic roof key block *B* above the coal wall of the lower section of the large structure, and the thickness and length of key block *B*. Accordingly, precracking the roof by artificial intervention could prevent the generation of the cantilever beam structure formed by incomplete collapse of the direct roof. In contrast, artificial precracking of the roof could increase the height of the collapsed area, and the collapsed rock in the range of the height of the cutoff top could result in key block *B* touching the gangue and forming a stable articulated structure as early as possible, as shown in Fig. 17b, thus decreasing the time for the collapsed area to be filled with air and reducing the sliding and sinking space for key block *B* in the range of the large structure. Accordingly, the stress environment of the enclosing rock of the small structure can be optimized.

In accordance with the abovementioned ideas of reducing the time and narrowing the space, the authors’ research team developed the method of dense drilling holes to release the pressure in the roof of a coal mine to protect the roadway^57,58^. The proposed method suggests that dense drill holes can be arranged along the roof on the side of the coal pillar to construct a discontinuous weak face for adjusting the lateral cantilever structure formed after mining the last working face, improving the stress state of the coal pillar and the coal wall of the lower working face and ensuring the stability of the coal pillar and the roadway during mining of the next workface. In the intensive drilling and pressure relief technology for coal mine roofs explored in this study, intensive drilling construction is completed outside the effect range of the workface oversupport pressure, which is usually minimized under the effect of the mining stress in the workface, so the weakening degree of the strength of the roof rock strata after intensive drilling is relatively low. When the effect range of the workface oversupport pressure enters the area of intensive drilling construction, the horizontal stress around the circular hole is redistributed under the effect of the mining stress, which leads to further expansion of the distribution range of the plastic area. In this process, the rock mass around the borehole undergoes progressive destruction along the direction of deviation from the center of the circular hole^58^, which causes a reduction in the strength of the top plate rock layer and thus promotes the timely collapse of the rock mass in the range of the roof-cut height and filling of the void area, early touching of the gangue and formation of a stable load-bearing structure.

The advantage of coal mine roof intensive drilling pressure relief technology is that it should only involve the construction of a series of intensive drilling holes in advance in the area outside the effect range of the overhead bearing pressure in the workface. Moreover, it is not required to conduct blasting or hydraulic fracturing, which involves a simple technological process, low equipment requirements, and large adaptation scope. Furthermore, compared with the stress wave and stress concentration generated by blasting and stress perturbation formed by hydraulic fracturing, the disturbance of the stress on the original rock resulting from the intensive drilling holes before the effect of the overhead bearing pressure in the workface is significantly low. With increasing stress perturbation, the surrounding rock of the roadway is not violently deformed, or coal rock dynamics are not induced. Thus, safety can be ensured. However, its construction quantity is large and the cost is relatively high.

### Design method for key parameters of dense drilling roof cutting and pressure relief

The process for determining the key parameters of dense drilling roof cutting and pressure relief technology requires the integration of theoretical analysis, numerical simulation, and field tests. Ultimately, through multi-parameter optimization, the critical parameters for dense drilling roof cutting technology are established. The details are as follows:

1. Determination of borehole diameter.

Based on the principle of large and small structures of the surrounding rock, the mechanical mechanism of stress correspondence around circular holes is analyzed. The borehole diameter directly affects the stress distribution around the hole and the extent of the plastic zone. Numerical simulation or theoretical calculations are used to analyze the distribution characteristics of the plastic zone around circular holes of different diameters. Research indicates that the stress concentration factor around a circular hole is related to its diameter. By comparing the disturbance effects of different diameters on the surrounding rock, the borehole diameter that can effectively weaken the roof while maintaining the stability of the borehole wall is selected. This ensures effective pre-splitting of the roof while guaranteeing that the drilling process does not cause instability in the surrounding rock.

(2) Determination of borehole spacing.

Based on the stress correspondence mechanism and the distribution characteristics of the plastic zone around circular holes of different diameters, the interaction between adjacent boreholes is analyzed. Numerical simulation software (e.g., UDEC) is used to simulate the stress distribution and plastic zone overlap between adjacent boreholes. The interference effects of different spacing on the stress field of adjacent holes are examined. Research indicates that as the spacing decreases, the plastic zones of adjacent holes begin to interconnect, forming a continuous weak plane. The optimal borehole spacing is determined to ensure the formation of interconnected fractures between adjacent holes (i.e., “interconnected plastic zones”) to guide roof caving, while avoiding excessive drilling to reduce engineering costs and impact on the surrounding rock.

(3) Determination of borehole angle.

The angle of the borehole determines the orientation of the fracture surface and the effect of roof cutting. Different borehole angles result in varying directions of stress release and caving characteristics of the roof strata. The effects of different roof cutting angles on the caving characteristics of the lateral cantilever structure of the working face and the distribution of mining pressure in the roadway are studied. By simulating the caving patterns of the roof under different angles (e.g., vertical, inclined), the impact of the angle on goaf filling and roadway stress reduction is analyzed. The goal is to find the borehole angle that most effectively promotes timely roof caving and filling of the goaf, thereby minimizing stress in adjacent roadways.

(4) Determination of roof cutting height.

The roof cutting height determines the extent of the cantilever structure to be cut and the horizon of roof caving. An excessively low cutting height may fail to achieve sufficient pressure relief, while an excessively high cutting height could affect the stability of the overlying strata. Based on specific geological conditions, a numerical model of the working face (e.g., UDEC) is established. The caving characteristics of the roof and the distribution of mining pressure in the roadway under different cutting heights are simulated. The analysis considers how the roof, after caving, fills the goaf and forms a stable load-bearing structure upon compaction. The optimal roof cutting height is determined to ensure significant roof caving and effective goaf filling, thereby maximizing the stability of the adjacent roadway and coal pillar.

The mechanical conditions and input parameters selected in the theoretical analysis and numerical simulation process have a critical influence on understanding the pressure relief mechanism of roof cutting with densely drilled holes. This study, based on the specific engineering case of the Chahasu Coal Mine, analyzes the effects of borehole diameter, borehole spacing, borehole angle, and borehole depth on the pressure relief effect of roof cutting with densely drilled holes. Combined with the onsite strata behavior patterns, a comparative analysis of the pressure relief and roadway protection effects of densely drilled holes is conducted. However, the establishment of the mechanical model and numerical calculation model in this study is limited to specific engineering conditions. Therefore, the findings of this research can provide a useful reference for engineering practices of roof cutting, pressure relief, and roadway protection under similar conditions.

## Conclusion

Based on the large–small structure theory of surrounding rock, this study investigates the stress distribution and plastic zone evolution around dense drilling holes under different parameters using theoretical analysis, UDEC numerical simulation, and field tests at the 31,315 working face of the Chahasu Coal Mine. The main conclusions are as follows:

1. The tangential stress concentration factor *K* around a circular hole increases with the horizontal stress coefficient *λ* and hole diameter *D*. Under static pressure, the plastic zone range 2*Rp* is approximately 1.22*D*–1.64*D*; under mining-induced stress, it expands to 4.21*D*–5.33*D*. Accordingly, the recommended spacing for intensive drilling is *L* = 8–10*D*.
2. The height and angle of roof cutting significantly affect the fracture characteristics and goaf filling of the lateral roof. Moderate increases in cutting height and angle promote timely roof collapse and the formation of a stable bearing structure. However, excessive cutting angles may induce an unfavorable cantilever structure. For the 31,315 working face conditions, a cutting height < 15 m and angle < 15∘are suggested to achieve effective pressure relief.
3. Industrial tests in the 31,317 roadway show that intensive drilling pre-cracking effectively mitigates floor heave, reduces stress concentration, and enhances roadway stability. The pressure relief effect improves with decreasing drilling spacing, confirming the feasibility of the proposed technique for roadway protection.

## Data Availability

The datasets used and/or analysed during the current study available from the corresponding author on reasonable request.
